# The application of prophylactic balloon occlusion of the internal iliac artery for the treatment of placenta accreta spectrum with placenta previa: a retrospective case-control study

**DOI:** 10.1186/s12884-020-03041-4

**Published:** 2020-06-08

**Authors:** Ying Peng, Lai Jiang, Cheng Peng, Dabao Wu, Ling Chen

**Affiliations:** Department of Obstetrics and Gynecology, First Affiliated Hospital of University of Science and Technology of China (Anhui Provincial Hospital, China), Hefei, 230001 Anhui Province China

**Keywords:** Placenta accreta spectrum, Embolisation of the internal iliac artery, Caesarean section

## Abstract

**Background:**

Severe obstetric haemorrhage caused by placenta accreta spectrum (PAS) results in significant maternal morbidity and mortality. The effectiveness of prophylactic balloon occlusion of the internal iliac artery in PAS patients remains controversial. Therefore, we conducted a retrospective case-control study to investigate the clinical effectiveness of this treatment.

**Methods:**

The clinical data of 104 patients with PAS complicated with placenta previa who delivered by caesarean section between January 2016 and January 2019 were collected, and the patients were divided into two groups. The study group (48 cases) underwent internal iliac artery preset balloon occlusion before caesarean section and uterine artery embolisation according to the bleeding status after surgery, while the control group (56 cases) did not undergo internal iliac artery preset balloon occlusion before caesarean section.

**Results:**

The operation and hospitalisation times in the study group were longer than those in the control group. Additionally, the hysterectomy rate in the study group was significantly higher than that in the control group. No significant differences in blood loss, blood transfusion volume, urinary system injury, postoperative ICU transfer rate, or neonatal scores were identified between the groups. Among the patients without invasive placenta (placenta increta and percreta), blood loss was lower in the study group, and the caesarean hysterectomy rate did not significantly differ between the groups. Among the patients with invasive placenta, blood loss and the caesarean hysterectomy rate did not significantly differ between the groups. The risk of hysterectomy in the study group was related to invasive placenta penetration, a large area of placental invasion, or abnormal vascular filling. One patient in the study group had a thrombus in the left lower extremity artery.

**Conclusions:**

Balloon occlusion of the internal iliac artery is effective for haemostasis of placenta previa in the absence of invasive placenta. For patients with invasive placenta, especially placenta percreta, a large area of placental invasion or abnormal vascular filling suggests the need for hysterectomy. The risks of the prophylactic use of internal iliac artery balloon occlusion include vascular injury and thrombus formation.

## Background

Placenta accreta spectrum (PAS) was first described by Chattopadhyay et al. [1] in 1993 as the main cause of obstetric haemorrhage and perinatal hysterectomy [2, 3]. PAS is classified into three subtypes depending on the depth of placental invasion: abnormally adherent placenta (placenta adherenta or accreta), abnormally invasive placenta (increta), and abnormally invasive placenta (percreta) [4, 5]. In recent years, due to increases in the caesarean section rate and uterine cavity operation rate, the incidence of PAS has increased, and the incidence of related postpartum haemorrhage has also increased [6]. Uncontrollable postpartum haemorrhage can occur when PAS is present in combination with placenta previa [7–9]. Caesarean section is the main treatment method for PAS, and haemorrhage control during the operation is key for successful treatment. When traditional methods such as uterine cavity compression [10, 11], uterine cavity water sac compression [12, 13], uterine compression suturing [14, 15], or uterine artery or internal iliac artery ligation [16, 17] cannot control massive haemorrhage, hysterectomy is required [18, 19]. However, hysterectomy results in physiological damage to the patient and imposes a mental burden on parturients and their families. Preventive placement of balloons in the bilateral internal iliac arteries before caesarean section can reduce uterine artery pressure and intraoperative blood loss during balloon inflation, thus temporarily blocking the main blood supply of the uterus, helping to expose the visual field, shortening the operation time during surgery, and leading to opportunities for timely adjustments to the operative plan during surgery. However, no consensus is available on its safety and effectiveness. In this paper, the clinical data of 104 patients with PAS complicated with placenta previa who underwent caesarean section and were admitted to our hospital between January 2016 and January 2019 were collected to investigate the clinical effect of internal iliac artery embolisation in PAS.

## Methods

### Data

The clinical data of 104 patients with PAS who underwent caesarean section and were admitted to our department from January 2016 to January 2019 were collected. The typical B-ultrasound findings were based on the detection of at least two of the following characteristics: 1) loss/irregularity of the hypoechoic area between the uterus and placenta depicted as a ‘retroplacentar clear zone’; 2) thinning/interruption of the uterine serosa-bladder wall interface; 3) myometrial thickness (< 1 mm); 4) turbulent placental lacunae with high-velocity flow (> 15 cm/s); 5) the presence of increased vascularity of the uterine serosa-bladder wall interface; and 6) loss of the vascular arch parallel to the basal plate and irregular intraplacental vascularisation [20–23]. All patients underwent caesarean section.

### Inclusion criteria

The inclusion criteria were: 1) availability of B-ultrasound examination and/or pelvic MR plain scans; 2) singleton pregnancies; 3) gestational age ≥ 28 weeks; 4) a history of caesarean section; and 5) a desire to continue giving birth. The exclusion criteria were: 1) serious internal or surgery-related disease; or 2) a tendency for bleeding. All patients were fully informed of the study and surgical procedure by their doctors and signed informed consent forms, and the research was approved by the Biomedical Research Ethics Committee of our hospital.

Area and weighing methods were used to measure bleeding volume. The area method was used to estimate the amount of blood loss according to the wet gauze blood area. For the weighing method, a pre-weighed towel was spread under the buttocks of the parturient. The wet weight of the dressing (g) was measured after bleeding, and the dry weight of the dressing (g)/1.05 (blood specific gravity g/ml) was measured before bleeding.

### Observation group

#### Study group

Balloon occlusion of the bilateral internal iliac arteries was performed. The procedure began with routine disinfection, followed by towel spreading and the administration of local anaesthesia with 2% lidocaine. After successful puncture through the right and left femoral arteries using the Seldinger technique, 7F arterial catheter sheaths were placed on the left and right sides. A balloon catheter was inserted into the appropriate position of the main trunk of the common iliac artery on the same side by means of guide wire anchoring and catheter exchange techniques. A small amount of contrast agent was injected to confirm that the balloon catheter was located in the internal iliac artery, and the position of the balloon catheter and arterial sheath was fixed. Immediately after delivery, the balloon was filled to block blood flow. According to the amount of intraoperative haemorrhage and placental accretion of the parturient, pulse embolisation, promotion of uterine contraction, and other treatments were carried out. If no active bleeding was evident, the bilateral internal iliac artery balloon was removed after caesarean section.

#### Control group

Caesarean section was performed routinely. After delivery, uterine massage, surface suturing after placental stripping for haemostasis, administration of a uterine contraction agent, and gauze packing in the uterine cavity were performed meticulously according to the specific conditions of the patient during the operation. According to the specific condition of the patient, total hysterectomy or subtotal hysterectomy was performed when necessary.

### Statistical analysis

EXCEL 2003 (Microsoft Corp., Redmond, WA) was used for data entry and sorting. SPSS 19.0 (IBM Corp., Armonk, NY) was used for data analysis. Age, the number of gestational weeks, the number of pregnancies, the number of caesarean sections, operation time, intraoperative bleeding volume, blood transfusion volume, postoperative hospitalisation length, new-born APGAR score, and other measurement data were compared by means of independent two-sample t-tests. A chi-square test was used to compare the invasive placenta rate, the hysterectomy rate, the placenta percreta rate, the rate of a placental invasive area > 1/2, the rate of abnormal vascular filling, etc. The puerperal infection rate, urinary system injury rate, and intensive care unit (ICU) occupancy rate were compared using Fisher’s exact test, with *P* < 0.05 indicating a significant difference.

## Results

The patients were divided into a study group and control group according to whether they received balloon occlusion of the internal iliac artery. The study group included 48 patients ranging in age from 22 to 41 years, with an average age of 32.08 ± 3.94 years. The number of gestational weeks ranged from 28 + 6 to 39 + 4 weeks, with an average of 35 ± 2 weeks. The patients had been pregnant 1 to 5 times, with an average of 2.81 ± 1.30 pregnancies. The average number of caesarean sections was 1.19 ± 0.45. There were 28 cases with invasive placenta in the study group. The control group comprised 56 patients aged 26 to 44 years, with an average age of 33.46 ± 4.53 years. The number of gestational weeks ranged from 30 + 4 to 39 + 2 weeks, with an average of 36 ± 2 weeks. The patients had been pregnant 1 to 7 times, with an average of 3.23 ± 1.53 pregnancies. The average number of caesarean sections was 1.18 ± 0.39, and there were 25 cases with invasive placenta. No significant differences in age, the number of gestational weeks, the number of pregnancies, the number of caesarean sections, or the number of invasive placenta events were identified between the two groups (*P* > 0.05), as shown in Table 1.

**Table 1:** Comparison of general data between the control group and study group

	Control group	Study group	t (X2)	P
N	56	48		
Age, years (mean ± SD)	33.46 ± 4.53	32.08 ± 3.94	1.65	0.103
Gestational weeks (mean ± SD)	36.05 ± 1.66	35.57 ± 1.97	1.36	0.177
Number of pregnancies (mean ± SD)	3.23 ± 1.53	2.81 ± 1.30	1.54	0.138
Number of caesarean sections (mean ± SD)	1.18 ± 0.39	1.19 ± 0.45	−0.11	0.913
Invasive placenta [N (%)]	25 (44.64%)	28 (58.33%)	1.938	0.164

*SD* Standard deviation.

We compared the two groups in terms of the operation time, intraoperative bleeding volume, blood transfusion volume, postoperative hospitalisation length, hysterectomy rate, urinary system injury rate, puerperal infection rate, ICU admission rate, and new-born APGAR score. We found that the operation time, postoperative hospitalisation time, and hysterectomy rate were significantly higher in the study group than in the control group (operation time: 158.44 ± 57.31 min vs. 104.2 ± 46.22 min; postoperative hospitalisation time: 7.02 ± 3.77 days vs. 5.70 ± 2.57 days; and hysterectomy rate: 29.2% vs. 12.5%, *P* < 0.05). No significant differences were found between the two groups in terms of the intraoperative haemorrhage volume, blood transfusion volume, urinary system injury rate, puerperal infection rate, ICU admission rate, and 1-min or 5-min APGAR score, as shown in Table 2.

**Table 2:** Comparison of maternal surgery and neonatal scores between the control group and the study group

	Control group	Study group	t (X2)	P
N	56	48		
Operation time, min (mean ± SD)	104.20 ± 46.22	158.44 ± 57.31	−5.34	0
Intraoperative bleeding volume, ml (mean ± SD)	1108.04 ± 1008.32	1504.17 ± 1123.44	−1.9	0.061
Blood transfusion volume, ml (mean ± SD)	970.54 ± 1083.21	1352.08 ± 1211.03	−1.70	0.093
Postoperative hospitalisation length, days (mean ± SD)	5.70 ± 2.57	7.02 ± 3.77	−2.06	0.043
Hysterectomy [N (%)]	7 (12.5%)	14 (29.2%)	4.45	0.035
Urinary system injury	3 (5.36%)	4 (8.33%)		0.701*
Puerperal infection [N (%)]	0 (0.0%)	2 (4.2%)		0.211*
ICU admission [N (%)]	3 (5.36%)	4 (8.33%)		0.701*
1-min APGAR score, mean ± SD	8.77 ± 1.25	8.58 ± 1.7	0.64	0.526
5-min APGAR score, mean ± SD	9.57 ± 0.81	9.35 ± 1.55	0.92	0.362

Note: ^*^Fisher’s exact test was used for comparisons

*ICU* Intensive care unit, *SD* Standard deviation.

Among the patients without invasive placenta, the amount of bleeding in the study group was significantly lower than that in the control group. No hysterectomies were performed in the 18 patients who underwent balloon occlusion. Among the 31 patients who did not undergo balloon occlusion, the hysterectomy rate was 3.23%. The difference was not statistically significant, as shown in Table 3.

**Table 3:** Comparison of the patients without an invasive placenta

	No balloon occlusion group	Balloon occlusion group	t (X2)	P
N	31	20		
Intraoperative bleeding volume, ml (mean ± SD)	887.10 ± 311.71	690.00 ± 226.88	2.438	0.018
Hysterectomy [N (%)]	1 (3.23%)	0 (0.00%)		0.658*

Note: ^*^Fisher’s exact test was used for comparisons

*SD* Standard deviation.

Among the patients with an invasive placenta, the differences in the mean blood loss and hysterectomy rate between the two groups were not statistically significant as shown in Table 4.

**Table 4:** Comparison of the patients with an invasive placenta

	No balloon occlusion group	Balloon occlusion group	t (X2)	P
N	25	28		
Intraoperative bleeding volume, ml (mean ± SD)	1692.00 ± 1280.60	2085.71 ± 1148.50	−1.18	0.243
Hysterectomy [N (%)]	6 (24.00%)	14 (50.00%)	3.8	0.051

*SD* Standard deviation.

**Fig. 1:**
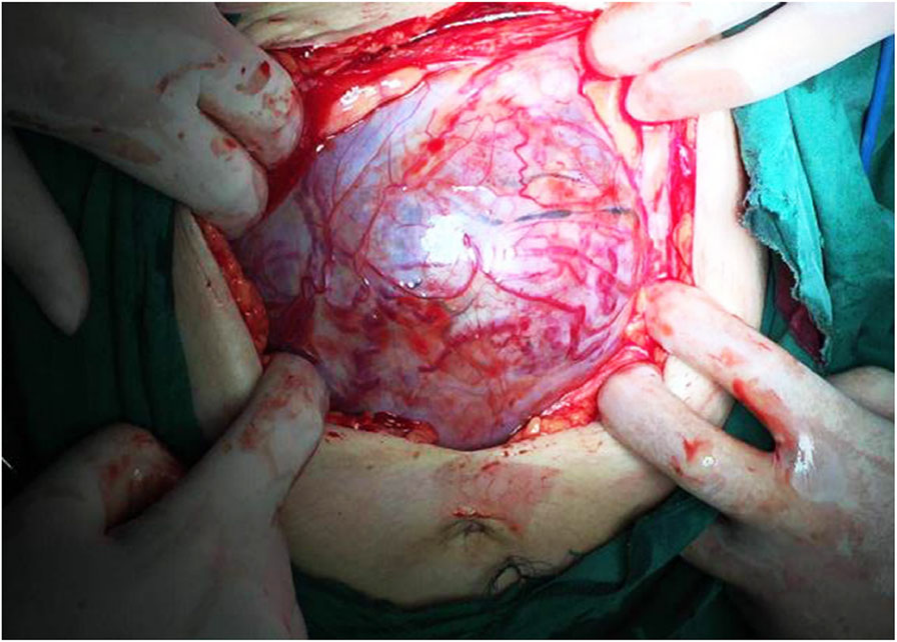
At gestational week 36^+ 2^, total placenta previa with increased vascularity of the uterine serosa-bladder wall interface, myometrial thinning of the anterior wall and penetration of the placenta close to the serous layer is observed

**Fig. 2:**
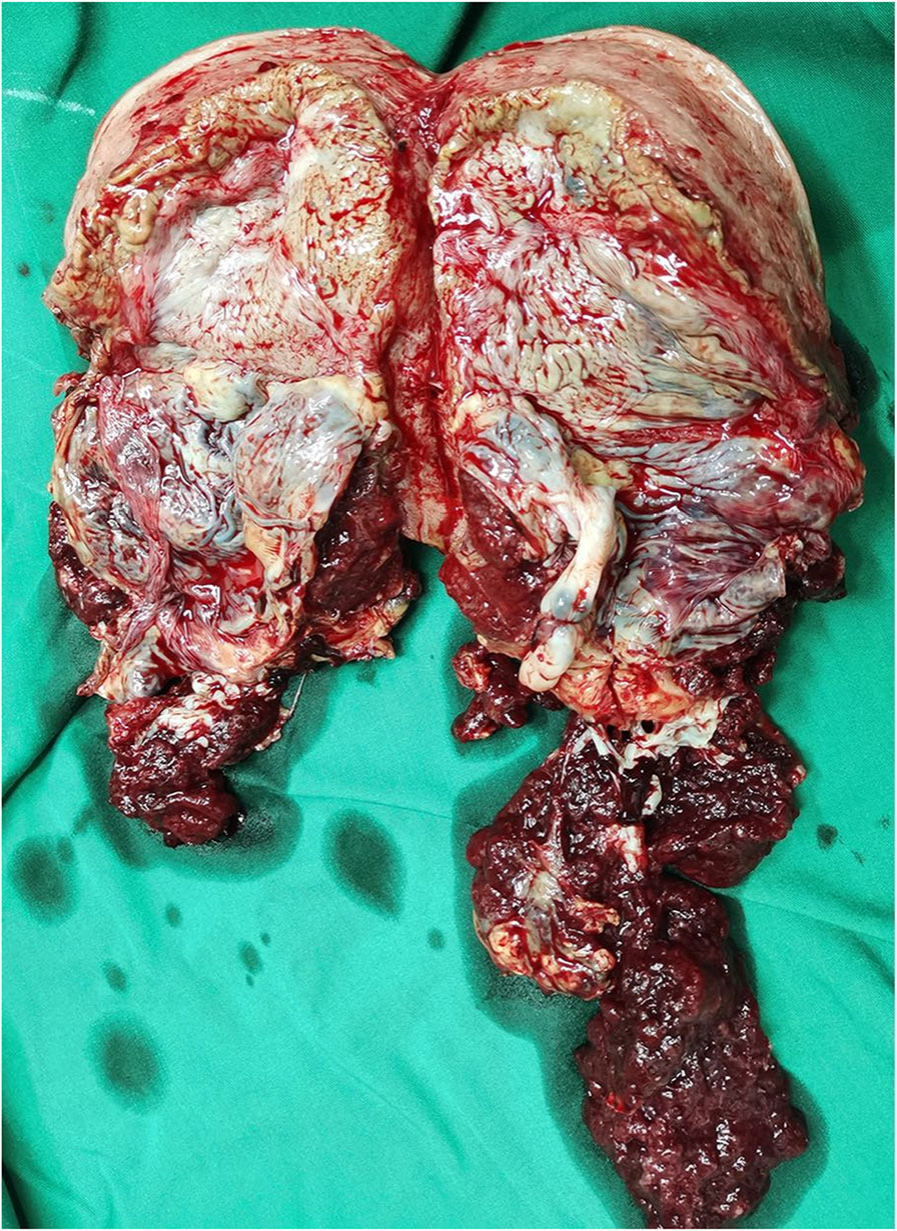
At gestational week 35^+ 2^, total placenta previa with placenta-penetrating invasion and a large invasive area is observed. No boundary between the uterus and placenta. Most of the placenta is located in the posterior wall of the uterus, where the myometrium is very thin

**Fig. 3:**
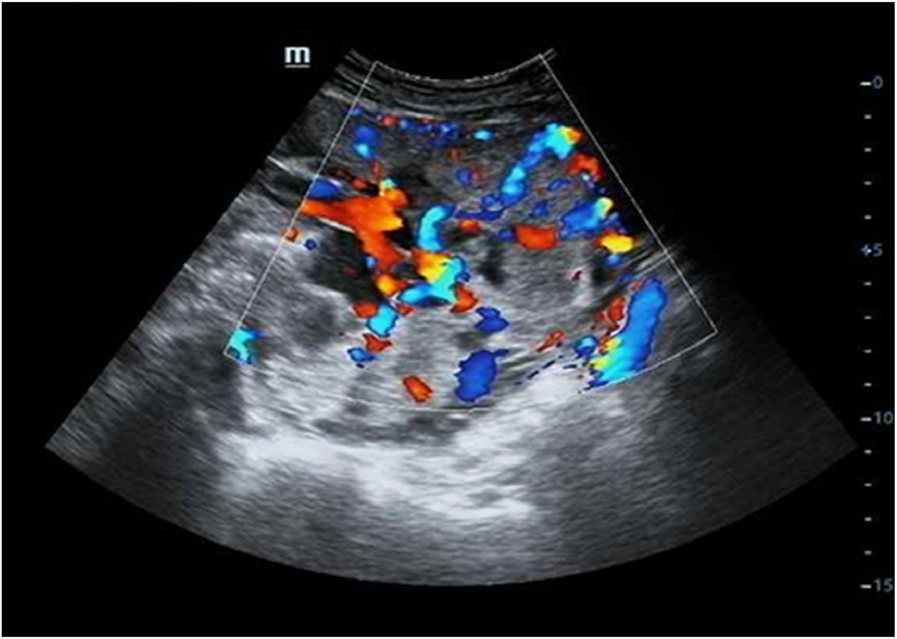
Ultrasound (US) imaging at gestational week 37 reveals total placenta previa with multiple vascular lacunae within the placenta. The placenta in the anterior wall reaches the serous layer of the uterus, and thinning of the uterine serosa-bladder wall interface is observed. Power doppler showing increased vascularity

**Fig. 4:**
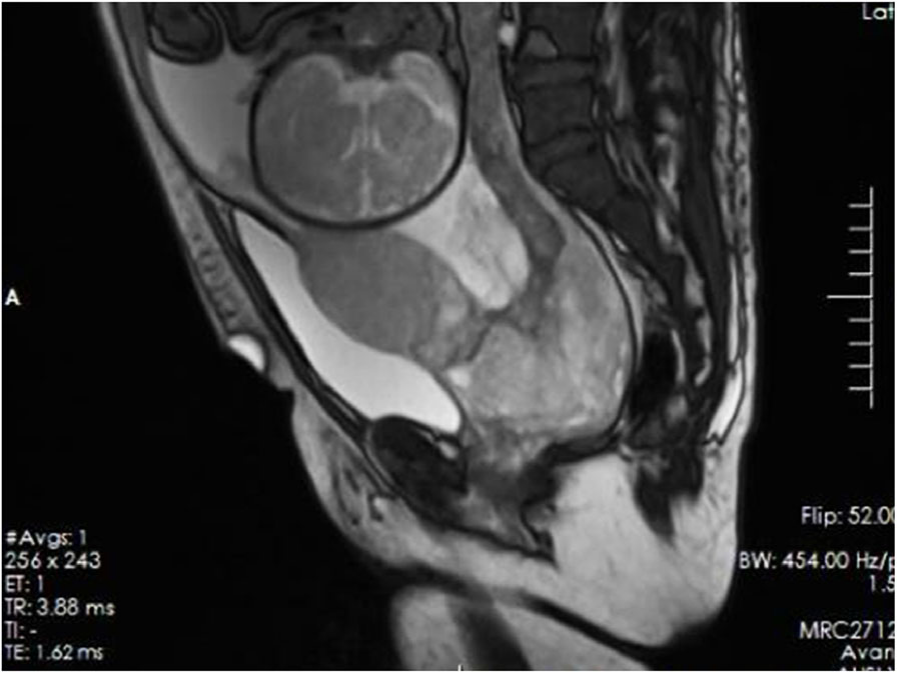
Magnetic resonance imaging (MRI) at gestational week 36^+ 5^ reveals total placenta previa. The placenta is located mainly on the anterior side. A T2 dark band, uterine bulging, myometrial thinning of the anterior wall and myometrium loss between the placenta and bladder wall are observed. Multiple linear processes that had invaded into the myometrium are observed

We compared the patients in the study group who did and did not undergo hysterectomy in terms of invasive placenta penetration (as shown in Fig. 1), a placental invasive area > 1/2 (as shown in Fig. 2), abnormal vascular filling (as shown in Figs. 3 and 4) and the number of caesarean sections. The rates of invasive placenta penetration, a placenta invasive area > 1/2 and abnormal vascular filling in the hysterectomy group were significantly (*P* < 0.05) higher than those in the unresected group (85.7, 92.9, and 85.7% vs. 11.8, 26.5, and 23.5%, respectively) as shown in Table 5.

**Table 5:** Patients with and without hysterectomy in the study group

Indicators	Unresected uterus	Hysterectomy	t (X^2^)	P
N	34	14		
Invasive placenta penetration [N (%)]	4 (11.8%)	12 (85.7%)	24.40	0.000
Placenta invasive area > 1/2 [N (%)]	9 (26.5%)	13 (92.9%)	17.60	0.000
Abnormal vascular filling [N (%)]	8 (23.5%)	12 (85.7%)	15.78	0.000
Previous caesarean section (mean ± SD)	1.21 ± 0.48	1.14 ± 0.36	0.44	0.661

*SD* Standard deviation.

## Discussion

The purpose of this study was to investigate the clinical effect of internal iliac artery balloon occlusion in patients with PAS and placenta previa. Postpartum haemorrhage is one of the main causes of maternal death [24, 25]. PAS often causes uncontrollable bleeding. Balloon occlusion of the internal iliac artery can temporarily block the internal iliac artery, effectively reduce the blood supply of the uterine artery and save the time available for the actual surgery. Although some studies have shown that preoperative prophylactic arterial catheterisation can reduce blood loss and transfusion demands in patients with placental invasion and help maintain fertility, its effectiveness in PAS remains controversial. Gulino et al. [26] reported 37 cases of placenta accreta, including 16 cases in a balloon group and 21 cases in a non-balloon group. Bleeding, the blood transfusion volume, and the rate of hysterectomy were lower in the balloon group than in the non-balloon group. Carnevale et al. [27] also concluded that preventive internal iliac artery balloon occlusion can reduce the amount of bleeding and blood transfusion in patients with placenta accrete after caesarean section and that this method is safe. However, preventive internal iliac artery balloon occlusion is not effective in all cases [28–32].

In this study, balloon placement did not reduce the amount of intraoperative bleeding or the rate of hysterectomy. The operation time and postoperative hospital stay were longer in the study group than in the control group. Further grouping showed that the bleeding volume of patients without an invasive placenta decreased after the balloon placement, supporting the haemostasis effect of internal iliac artery balloon occlusion for non-invasive placenta, whereas the bleeding volume effect in the patients with an invasive placenta was not obvious.

Shrivastava et al. [31] believe that the failure of internal iliac artery balloon occlusion to reduce blood loss may be due to excessive uterine blood flow during pregnancy and extensive intrapelvic vascular anastomosis. In addition to uterine artery support, the blood supply of the uterus also includes the obturator artery, ovarian artery and femoral artery [33, 34]. In PAS with placenta previa, the placenta is mostly located in the lower part of the uterus, the cervix and the upper part of the vagina, where many abnormal vascular anastomotic branches exist [35, 36]. Through the extensive collateral circulation between the arteries in the pelvis, the branches originating from other blood vessels (i.e., the external iliac artery or the femoral artery) can quickly compensate for the occluded arteries of the uterus, which is not conducive to completely blocking uterine blood flow. The more invasive the placenta, the more obvious the changes in the blood vessels of the placenta [37]. Therefore, internal iliac artery balloon occlusion may not completely block the uterine blood supply of an invasive placenta.

In the study group, high-risk factors for hysterectomy were invasive placenta penetration, placenta invasive area > 1/2 and abnormal vascular filling. The intra- and postsurgical outcomes of women affected by PAS are directly related to the depth and topography of placental invasion, and those affected by placenta percreta are at a higher risk of morbidity [38, 39]. Placenta percreta can invade through the myometrium and serosa, causing disastrous blood loss. Maternal mortality is as high as 10% [40]. The expected treatment failure rate for placenta percreta patients is 44% (8/18) [41]. The placental invasive area is large, the blood vessels on the uterine surface are characterised by hyperplasia, bleeding is increased, and the rate of hysterectomy is increased. The development of intrapelvic parasitic arteries in the gravid uterus is hypothesised as the main cause of bleeding [34]. In PAS, abnormal blood vessels can be found around the uterus and bladder. If the bladder and incision are not correctly identified, vascular damage and serious bleeding may occur. The lack of decidua in PAS leads to the highly invasive phenotype of extravillous trophoblasts as well as an imbalance and an increase in blood vessels at the site of invasion. The structure of blood vessels in the placental bed changes substantially, the distribution of blood vessels becomes uneven, and the size is uneven [42, 43]. In this case, even if a balloon is used, efforts to preserve the uterus for the patient may fail. The patients in the study group who underwent hysterectomy at our hospital exhibited abnormal blood vessel filling, and all these patients had placenta percreta. Five of these cases had concomitant bladder invasion, and the amount of bleeding was not reduced after intervention. Four cases had urinary system injury during the operation. Adjusting the treatment plan during the operation in a timely and effective manner in such patients remains an important topic of discussion. Abdominal aortic balloon occlusion has reportedly been used in patients with abnormalities of placental invasion and placenta accreta [44, 45].

Although the internal artery balloon occlusion procedure to reduce bleeding during caesarean delivery shows some effectiveness, its side effects on patients should be considered. Balloon occlusion of the internal iliac artery requires bilateral femoral artery puncture and the use of two balloons, which increases the exposure time and trauma to patients during the operation and requires a fixed body position after the operation. The complications after vascular embolisation also remain controversial and may include ischaemic necrosis of the lower limbs, thrombosis of the internal iliac artery, haematoma at the puncture site, ischaemia-reperfusion injury of tissues and organs and acute renal failure, among others. Iliac artery thrombosis has been reported to be the most common complication, with a risk of approximately 5% [44, 46, 47], which may be due to changes in maternal blood flow, vascular injury during puncture and the hypercoagulable state of blood during pregnancy [46]. One patient developed lower limb thrombosis on the first day after balloon occlusion of the internal iliac artery in our hospital after internal iliac artery embolisation. The patient underwent left femoral artery incision and embolisation + femoral artery artificial vessel bypass. The size of the balloon, the size of the arterial sheath and the time of balloon occlusion are important factors. If the balloon is too large, arterial injury may occur. If the balloon occlusion time is too long, thrombosis and reperfusion injury of the lower limbs may occur.

In addition, the foetal radiation exposure dose must be minimised. At the same exposure time [48], the X-ray dose of digital subtraction angiography (DSA) far exceeds the dose of fluoroscopy. Although this technology has been applied clinically for approximately 20 years, no consensus or data are available regarding the safe dose for the foetus, and further research and statistics are needed. Sun et al. [49] recommended a dose of 4.2 ± 1.9 mGy. To minimise radiation exposure, experienced interventional doctors should quickly insert the balloon into the internal iliac artery. During the follow-up of new-borns in our hospital, no complications caused by radiation were noted.

This study had certain limitations due to its retrospective and single-centre nature, its small sample size and possible selection bias. Thus, larger multi-centre studies are required**.**

## Conclusions

In conclusion, the preventive use of an internal iliac artery balloon is one method for the treatment of PAS patients as this method can control bleeding during caesarean section. However, the need to control complications related to balloon occlusion warrants consideration. Indications should be strictly controlled. The benefits of reducing blood loss must be balanced with the invasiveness of this procedure and the risk of maternal adverse events [50], and doctor-patient communication should be comprehensive.

## Data Availability

The data sets used and analysed during the current study are available from the corresponding author upon reasonable request.

## Abbreviations

PAS: Placenta accreta spectrum
MRI: Magnetic resonance imaging
DSA: Digital subtraction angiography
